# An Okinawan-Based Nordic Diet Leads to Profound Effects on Gut Microbiota and Plasma Metabolites Linked to Glucose and Lipid Metabolism

**DOI:** 10.3390/nu15143273

**Published:** 2023-07-24

**Authors:** Lokeshwaran Manoharan, Bodil Roth, Corinna Bang, Hans Stenlund, Bodil Ohlsson

**Affiliations:** 1 National Bioinformatics Infrastructure Sweden (NBIS), SciLifeLab, Division of Occupational and Environmental Medicine, Department of Laboratory Medicine, Lund University, SE-22100 Lund, Sweden; lokeshwaran.manoharan@nbis.se; 2 Department of Clinical Sciences Malmö, Lund University, SE-22100 Lund, Sweden; bodil.roth@med.lu.se; 3 Department of Internal Medicine, Skåne University Hospital, SE-20502 Malmö, Sweden; 4 Institute for Clinical Molecular Biology, Christian-Albrecht’s University, 24105 Kiel, Germany; c.bang@ikmb.uni-kiel.de; 5 Umeå Plant Science Centre (UPSC), Department of Plant Physiology, Umeå University, 90187 Umeå, Sweden; hans.stenlund01@umu.se

**Keywords:** anthropometry, Okinawan-based Nordic diet, metabolomics, glucose metabolism, gut microbiota, IL-18, lipid metabolism

## Abstract

Dietary interventions modify gut microbiota and clinical outcomes. Weight reduction and improved glucose and lipid homeostasis were observed after adopting an Okinawan-based Nordic diet (O-BN) in individuals with type 2 diabetes. The aim of the present study was to explore changes in metabolomics and gut microbiota during O-BN and correlate changes with clinical outcomes. A total of 30 patients (17 women), aged 57.5 ± 8.2 years, diabetes duration 10.4 ± 7.6 years, 90% over-weight, were included. Participants were provided an O-BN for 12 weeks. Before and after intervention, and 16 weeks afterwards, anthropometry and clinical data were estimated and questionnaires were collected, as well as samples of blood and stool. Plasma metabolomics were determined by gas- (GC-MS) or liquid- (LC-MS) chromatography-based mass spectrometry and fecal microbiota determination was based on 16S rRNA amplicons from regions V1–V2. During the intervention, weight (6.8%), waist circumference (6.1%), and levels of glucose, HbA1c, insulin, triglycerides, and cholesterol were decreased. Of 602 metabolites, 323 were changed for any or both periods; 199 (101 lipids) metabolites were decreased while 58 (43 lipids) metabolites were increased during the intervention. Changes in glucose homeostasis were linked to changes in, e.g., 1,5-anhydroglucitol, thyroxine, and chiro-inositol. Changes of microbe beta diversity correlated positively with food components and negatively with IL-18 (*p* = 0.045). Abundance differences at phylum and genus levels were found. Abundances of Actinobacteria, Bacteroidetes, Firmicutes, and Verrucomicrobia correlated with anthropometry, HbA1c, lipids, inflammation, and food. Changes in metabolites and microbiota were reversed after the intervention. The O-BN-induced changes in metabolomics and gut microbiota correspond to clinical outcomes of reduced weight and inflammation and improved glucose and lipid metabolism.

## 1. Introduction

The prevalence of type 2 diabetes is increasing in the world along with the increased body weight in the population [1]. Healthy lifestyle factors could potentially prevent many chronic diseases [2], hypothetically through changes in gut microbiota composition influencing inflammation and development of metabolic syndromes and diabetes [2,3,4]. High-fiber diets low in red meat, sucrose, and processed food lead to a more diverse and beneficial gut microbiota with improvements in clinical outcome, whereas Western diets with high animal fat and protein and low fiber content lead to increased amounts of pathogenic bacteria [2,5]. Dietary interventions in diabetes have been found to modulate microbiota with correlations to a more favorable metabolic profile and inflammatory markers [3,4,6]. Accordingly, probiotics have been shown to reduce levels of blood glucose and HbA1c and reduce insulin resistance [7].

Metabolomics reflects metabolism and is, therefore, dependent on dietary habits and physical activity [8]. Higher proportions of a specific macronutrient in the diet, e.g., fat, lead to an increased ability to oxidize the macronutrient primarily consumed [8,9]. Furthermore, the endogenous substrate concentrations increase after acclimating to a high-fat or high-carbohydrate diet [8,10].

Several diets have been examined regarding reduction of inflammation and glucose and lipid levels and improvement of cardiovascular health [11]. The traditional Okinawa diet characterized by increased content of fiber, fat, and protein based on raw food with minimal industrial processing has been modified to suit tastes and food components suitable for the Nordic population and called an Okinawan-based Nordic diet (O-BN) [12,13]. The meal composition is consistent with moderately low carbohydrate content [14]. The food is based on traditional Nordic raw food, e.g., whole grains, vegetables, fat fish, birds, fruits, berries, and nuts, with a reduction of dairy products, red meat, and processed meat, as well as sugar and white flour to have a diet with low glycemic index [13]. We have previously published how this O-BN led to marked clinical effects with reduction in weight, waist circumference, glucose levels, lipid levels, and blood pressure in alignment with endocrine effects [15]. One of the hypotheses for the marked improvements was that changes of the microbiota could influence the metabolism. The aim of the present study was therefore to analyze the composition of plasma metabolomics and gut microbiota, and correlate changes of these parameters with changes in anthropometric parameters, glucose and lipid homeostasis, and gastrointestinal symptoms.

## 2. Materials and Methods

### 2.1. Study Population and Design

**Figure 1:**
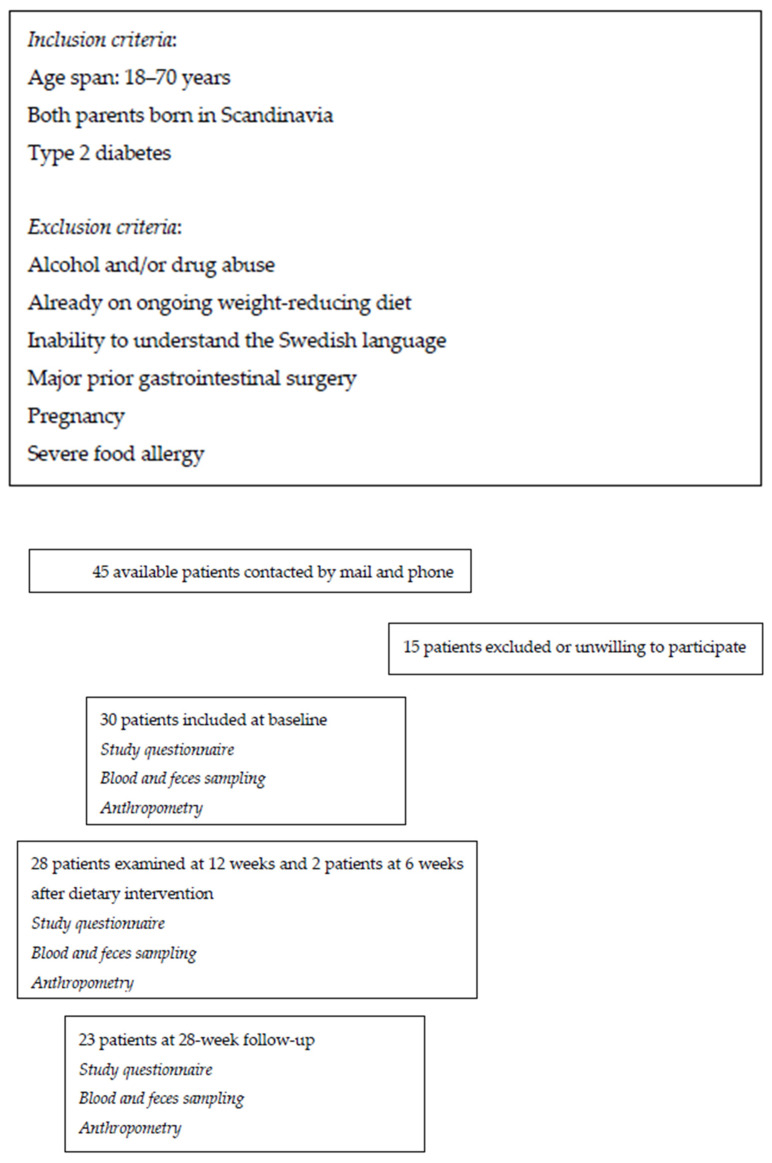
Flow chart of the recruitment process and study design.

A clinical prospective interventional trial with O-BN in type 2 diabetes (T2D) was performed at Skåne University Hospital, Malmö, Sweden, and conducted for 12 weeks (week-12), followed by a clinical follow-up after 16 weeks (week-28) with unrestricted diets. Patients with T2D, independently of BMI or anti-diabetic treatment regimen, aged between 18–70 years, were recruited at a primary healthcare center in the southernmost district of Sweden. Participants, being their own controls, were initially informed of the project through a letter, followed by a phone call one week later (Figure 1).

A detailed description of the study design, diet components, and methodology has been published [13]. Briefly, study data consisting of blood and feces sampling; assessments of anthropometric data; and completion of questionnaires was obtained at 3 separate visits: (1) at baseline before introduction of the diet (Baseline); (2) after 12 weeks on the O-BN (week-12); and (3) after 16 weeks on an unrestricted diet (week-28) (Figure 1). All participants were instructed by the nutritionist on how to prepare their breakfast. Food for lunch, dinner, and snacks was delivered to the homes of the subjects three times a week free of charge, along with written information and recipes for meal preparation. The participants were encouraged to maintain their regular physical activity habits throughout the intervention. Blood samples were drawn in the morning between 07:45 and 09:00 after a 10-h fast. Metabolic parameters were analyzed at once at the Department of Clinical Chemistry and plasma was stored at −80 °C until analyzed for metabolomics. Stool samples were collected at home in sterile tubes (Sarstedt, Numbrecht, Germany) and put in the freezer until they were brought to the lab. The samples were kept at –80 °C until extraction of microbial DNA. The study started on 2 February 2015 and ended on 18 September 2015.

### 2.2. Dietary Advice

The diet is based on the traditional Okinawa diet [12] but modified to suit tastes and food components suitable for the Nordic population [13]. The meal composition is consistent with moderately low carbohydrate content, one of four nutritional recommendations from the Swedish National Food Agency for patients with diabetes [14]. These recommendations are in line with international recommendations from the American Diabetes Association (ADA) and European Association for the Study of Diabetes (EASD) [16]. The contents of fiber, fat, and protein are increased, which lead to a bigger meal with more mastication and prolonged meal intake [17]. The food is based on traditional Nordic raw food, e.g., whole grains, vegetables, legumes, root crops, fat fish, birds, fruits, berries, and nuts, with minimal industrial processing. Furthermore, dairy products, red meat, and processed meat were limited, as well as sugar and white flour to have a diet with low glycemic index (Supplementary Table S1). The diet has a good nutritional supply compared to the recommendations, containing a mean calorie intake around 1900 kcal/day, including drinks, which is slightly lower compared to a traditional diet (Supplementary Table S2). The participants were allowed to eat three meals a day including breakfast, lunch, and dinner, and two snacks between meals consisting of a variety of fruits, berries, and seeds. Organic food items were preferred whenever possible. At the occurrence of cravings, the subjects were instructed to eat a third snack (e.g., carrots, boiled eggs, mackerel in tomato sauce, cottage cheese with berries) to avoid fast carbohydrates. The participants started the main meals with raw vegetables or green salad; 100 g at breakfast and 150 g at lunch and dinner, respectively.

The meals were planned at the kitchen of Igelösa Life Science Lab (Lund University) and delivered to the subjects regularly. The participants had to buy the breakfast food themselves, which consisted of porridge or fermented milk in combination with bread, depending on their ordinary breakfast.

The participants were encouraged to continue with supplements already consumed but were not allowed to introduce new supplements during the study period. At maximal one visit to a restaurant or another diet per week was allowed. Journeys had to be discussed with the investigators. Maximal intake of alcoholic beverages was set to 30 g ethanol/week.

The nutritionist had close contact with the subjects using email and telephone during the whole study to support them and enhance compliance as much as possible.

### 2.3. Questionnaires

#### 2.3.1. Study Questionnaire

A study questionnaire contained questions about ordinary lifestyle factors, sociodemographic factors, and medical history. Further, participants provided information on whether they already were on an ongoing weight-reducing diet, intake of dietary supplements, vitamins, probiotics, and use of antibiotics during the past 6 months.

#### 2.3.2. Nutrition Questionnaire

A nutrition questionnaire was used at recruitment to collect information on ordinary food habits and energy intake (Supplementary Material). This basic information was used to individually design the breakfast for the participants. The questionnaire was repeated at the end of the intervention and at follow-up. The participants had a good adherence to the diet [13]. The scores were calculated from the nutrition questionnaire, where the scores for fat mean the following: <40 is rather small amounts of fat, 40–50 is good, 51–71 is too much fat, and >71 is quite too much. The scores for fiber mean the following: 17–20 is very good, 12–16 is rather good, may be improved, and 6–11 is too low [18].

#### 2.3.3. Visual Analog Scale for Irritable Bowel Syndrome

Gastrointestinal symptoms were estimated using the validated VAS-IBS, measuring abdominal pain; diarrhea; constipation; bloating and flatulence; vomiting and nausea; psychological well-being; and the intestinal symptoms’ influence on daily life during the past 2 weeks on scales from 0–100, where 0 represents the absence of symptoms and 100 represents very severe symptoms. The scales are inverted from the original version [19].

### 2.4. Assessment of Clinical Variables and Anthropometry

The clinical investigation took place under identical conditions by experienced physicians. Diabetic complications were registered including autonomic neuropathy (sexual dysfunction, profound sweating, and orthostatic blood pressure), gastrointestinal dysmotility, levels of albuminuria (albumin/creatinine ratio), macroangiopathy, peripheral neuropathy (patellar and achilles tendon reflexes, vibration sense, and monofilament), and retinopathy (fundus photography).

### 2.5. Sampling and Chemistry Analyses

All samples consisted of whole blood drained into ethylenediaminetetraacetic acid (EDTA) glass tubes (BD Microtainer, Franklin Lakes, NJ, USA) or serum separation tubes (SST) with coagulation activator and gel (BD Microtainer). Blood was centrifuged at 3000 rcf for 10 min, and plasma was immediately cooled and stored in −80 °C until later metabolomics analyses. Interleukin-18 (IL-18) and adipokines in plasma; short-chain fatty acids (SCFA) in serum; and zonulin and calprotectin in feces were analyzed in-house as previously published [15]. Insulin in serum; glycated hemoglobin A1c (HbA1c) and leucocytes in blood; and C-reactive protein (CRP), glucose, liver enzymes, triglycerides, cholesterol, high-density lipoprotein (HDL), and low-density lipoprotein (LDL) in plasma were analyzed by standard methods at the Department of Clinical Chemistry [20].

### 2.6. Metabolomics

Plasma was examined at the Swedish Metabolomic Center, Umeå. The UHPLC-MS analysis was performed with an Infinity 1290 Agilent (Agilent Technologies, Santa Clara, CA, USA) ultra-high-performance liquid chromatograph coupled with tandem mass spectrometry (UHPLC-MSMS) as previously described in detail [21]. The GC-MS analysis was performed with Agilent 6890 GC equipment and a fused silica capillary column (10 m × 0.18 mm I.D.) with a chemically bonded 0.18 µm DB5-MS stationary phase (J&W Scientific, Folsom, CA, USA) [22].

The data pre-processing of both the UHPLC-MS data and the GC-MS data has previously been described [23]. The lipids were analyzed with respect to the chloroform fraction from the same extract as the ordinary metabolomics analysis [24]. There were in total 602 annotated metabolic features, comprising 565 unique annotations, for the combined LC-MS and GC-MS data. All metabolomics data were relative concentrations.

### 2.7. Gut Microbiota Analysis

DNA was extracted using the QIAamp DNA stool mini kit, automated on QIAcube. For this, 200 mg stool samples were transferred to 0.70 mm garnet bead tubes filled with 1.1 mL ASL lysis buffer (containing Proteinase K). Subsequently, bead beating was performed using a SpeedMill PLUS for 45 s at 50 Hz. Samples were then heated to 95 °C for 5 min and the following steps for DNA extraction were performed according to the manufacturer’s protocol.

Variable regions V1 and V2 of the 16S rRNA gene were amplified using the primer pair 27F-338R in a dual-barcoding approach [25]. DNA was diluted 1:10 prior to PCR, and 3 µL of this dilution was finally used for amplification. PCR products were verified using electrophoresis in agarose gel. PCR products were normalized using the SequalPrep Normalization Plate Kit (Thermo Fischer Scientific, Waltham, MA, USA), pooled equimolarly and sequenced on the Illumina MiSeq v3 2 × 300 bp (Illumina Inc., San Diego, CA, USA). Demultiplexing after sequencing was based on 0 mismatches in the barcode sequences. Cutadapt was used to trim the adapters and primers followed by filtering of low-quality sequence reads [26].

### 2.8. Statistical Analyses

A power analysis was performed where 18 subjects were needed to demonstrate clinically significant differences with 80% power at 5% significance level. To compensate for disappearance, we intended to recruit 25–35 subjects [13,27]. Two of the subjects interrupted the study after 6 weeks on diet, the data from which was calculated together with data from 12 weeks of intervention.

Basal characteristics were calculated by SPSS, version 25, using Student’s paired test, Wilcoxon’s test, Spearman’s test, or Fisher’s exact test. Values are presented as mean ± standard deviation (SD), median and interquartile range, or number and percentages.

**Figure 2:**
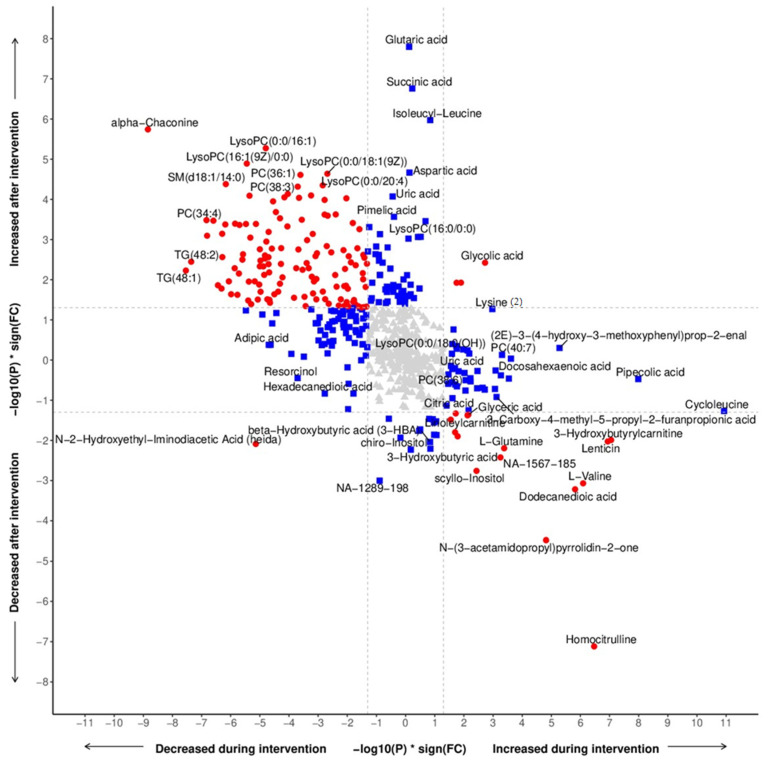
All the different metabolites measured using both GC-MS and LC-MS are plotted on this figure. The adjusted *p*-values (*p*) multiplied with their sign of fold-change (FC) (negative for downregulation and positive for upregulation) from the differential expression analysis are plotted on the axes. The differential expression analysis “during intervention (week-12 vs. baseline)” is plotted on the *x*-axis while the differential expression analysis “after intervention (week-28 vs. week-12)” is plotted on the *y*-axis. The metabolites that are differentially expressed in both comparisons are highlighted in red while the metabolites that are differentially expressed only in one comparison are highlighted in blue. The metabolites that are not differentially expressed in either comparison are in gray.

The preprocessed metabolomics data from the GC-MS, LC-MS, and lipidomics were analyzed separately. The data were first log2 transformed and visualized using a PCA. Further, the differentially expressed metabolites during intervention (week-12 vs. baseline) and after intervention (week-28 vs. week-12) were calculated using the ‘lmFit()’ function from the R package “limma” [28]. The variation due to the run-order of samples in the GC-MS data was corrected prior to the differential expression analysis. All the differential expression analyses from the different metabolomics data were combined for Figure 2.

The demultiplexed 16S samples from MiSeq were processed mainly with QIIME2 (v.2018.11) [29]. Within QIIME2, DADA2 [30] was used to predict the amplicon sequence variants (ASVs). Then, the taxonomy of ASVs were predicted using VSEARCH [31] together with the SILVA (v. 132) database [32]. The alpha and beta diversity metrics for all samples were calculated within QIIME2. Further statistical analyses were performed using ‘phyloseq’ [33] and ‘vegan’ [34] packages in R. The differentially abundant ASVs in the different sample groups were calculated using DESeq2 [35].

The alpha diversity was measured as Shannon–Weiner index, phylogenetic diversity, and Observed number of ASVs, and the beta diversity was measured using Bray–Curtis and UniFrac (both weighted and unweighted) [36]. Diversities were measured and visualized using the ‘phyloseq’ package. Wilcoxon’s *t*-test of comparing means was applied to test the alpha diversity from different groups and PERMANOVA was applied to beta diversity. Kruskal–Wallis test was applied for calculating different phylum abundances between different time points. Benjamini–Hochberg, or false discovery rate (FDR), was used to correct for multiple comparisons of abundances.

The correlation between the continuous physiological factors of the samples to the beta diversity were calculated using the ‘envfit’ function in the ‘vegan’ package and their correlation to alpha diversity and also phylum abundances were calculated using linear model fit function ‘lm()’ in R. The PERMANOVA analysis of the categorical variables on the beta diversity were performed using the ‘adonis’ function in the ‘vegan’ package.

A *p*-value < 0.05 was considered statistically significant.

## 3. Results

### 3.1. Clinical Characteristics

Forty-five patients were randomly selected for inclusion. In total, 30 (17 women) of these patients, aged 57.5 ± 8.2 years, diabetes duration 10.4 ± 7.6 years, were included in the study (Figure 1). Socioeconomic characteristics and the most common drugs and complications are shown in Table 1.

Breakfast was prepared by the participants themselves and was, therefore, the only component that differed between subjects (Supplementary Table S3). The fat score was decreased after both 12 and 28 weeks, whereas the fiber score was increased during the 12-week intervention, considering the total food intake (Supplementary Table S4).

Twenty-seven patients (90%) were over-weight, and 15 patients (50%) were obese at baseline [37]. Weight was reduced during the 12-weeks intervention, leading to 6.8% decrease in BMI and 6.1% reduced waist circumference (reduction in 29 and 28, respectively, of 30 individuals). A regression was observed in the follow-up with 1.8% in BMI and 0.8% in waist circumference (13 and 12, respectively, of 23 individuals). Both the systolic and diastolic blood pressure were decreased after the intervention. Fasting plasma levels of glucose, triglycerides, and cholesterol were decreased after 12 weeks, whereas HbA1c and the fasting insulin level were decreased both after 12 and 28 weeks (Supplementary Table S5). IL-18 was decreased during the study and correlated with the decrease in glucose (rs = 0.499, *p* = 0.005) and HbA1c (rs = 0.405, *p* = 0.026). The decrease in IL-18 tended to correlate with the reduced breakfast intake of g carbohydrates (rs = 0.352, *p* = 0.072) and energy percent (E%) of fiber (rs = 0.327, *p* = 0.096), as well as the calorie decrease (rs = 0.347, *p* = 0.076). The most profound effect on gastrointestinal symptoms were found on diarrhea and bloating and flatulence, which were improved throughout the study period, whereas abdominal pain only was decreased after 12 weeks. Constipation and nausea/vomiting were not affected by the diet. Psychological well-being was improved during the whole study period (Supplementary Table S6). 

### 3.2. Metabolomic Profiling

Obvious changes in the metabolic profiles were observed for the subjects undertaking the dietary intervention (i.e., the first 12 weeks of the study) (Supplementary Figure S1), with a clear beneficial health effect but also a return toward the baseline values for the subsequent post-intervention period (i.e., the 16 weeks after the intervention) (Figure 2). A total of 323 out of the 602 metabolic features showed a significant change for any or both periods. For the dietary intervention period, 199 metabolic features showed a significant decrease in levels, of which 101 were lipids, while 58 metabolic features showed a significant increase in levels, of which 43 were lipids. For the subsequent post-intervention period, 30 metabolic features showed significant decrease, of which none were lipids, while 182 metabolic features showed significant increase, of which 31 were lipids. The effect was obvious during the intervention, but afterwards the levels reverted toward baseline values, indicating that the subjects converted to their previous eating habits.

### 3.3. Metabolomics Related to Food Components

The consumption of potato (alpha-chaconine), fast-acting carbohydrates (glucose and ribitol), red meat (creatine, carnitine, glutamate, and tryptophan), and fat (most of the triacylglycerols/triglycerides and other lipids) was indicated to be drastically reduced during the intervention period while increasing for the subsequent post-intervention period. The consumption of lentil (lenticin), white meat (homocitrulline, glutamine), whole grains (valine), vegetables (glutamine, glyceric acid), and coconut products (dodecanedioic acid, scyllo-inositol) indicated the opposite trend, i.e., first increased, then decreased, levels. It became apparent that the subjects increased their consumption of fish and poultry during the intervention, which was mainly concluded from the significantly increased levels of docosahexaenoic acid (omega-3), homocitrulline, glutamine, lysine, 3-methylhistidine, asparagine, glycine, and threonine, all linked to white meat food sources. Also, the intake of whole grains, beans, legumes, vegetables, and fruits was increased during the intervention, linked to the significantly increased levels of pipecolic acid, coniferaldehyde, valine, xanthine, CMPF, citric acid, and beta-resorcylic acid (Figure 2).

### 3.4. Clinical Correlations

Increased levels of 1,5-anhydroglucitol (1,5-AG) (*p* = 0.018) and decreased levels of thyroxine (*p* = 0.020) were linked to lowered glucose levels (*p* < 0.001) during the intervention period. For the subsequent period, glucose levels increased (*p* = 0.031) while chiro-inositol levels decreased (*p* = 0.018). Most of the lyso-lipids showed strong significant reductions during the intervention and an overall strong significant increase for the subsequent post-intervention period. The higher levels of alpha-tocopherol at weeks 12 and 28 may reflect a change in the source of fat intake between the intervention and the follow-up.

During the intervention period, the subjects showed signs of starvation. The metabolic profiling data supported the overall reduction in calorie intake since mainly all the lipid/fat and dipeptide features showed a significant drop. The metabolites commonly associated with ketosis and fasting further showed a drastic reduction in food energy intake. The levels of 3-hydroxybutyrylcarnitine were clearly elevated during the intervention (*p* = 0.001). For the subsequent period, a significant drop in levels of 3-hydroxybutyrylcarnitine (*p* = 0.010), as well as ketone acids like 3-hydroxybutyrate (*p* = 0.006), acetoacetic acid (*p* = 0.017), and 2-ketobutyric acid (*p* = 0.019), was observed. It became evident that the intervention might lead to ketosis as an effect of lowering the intake of carbohydrates and fats while increasing the protein content, causing the levels of keto acids to drastically change. Cycloleucine was indicated as having the largest increase during the intervention period with a greater than two-fold change in abundance (*p* < 0.001) (Figure 2).

### 3.5. Hormones and Neurotransmitters

Cortisol (*p* = 0.003) and cortisone (*p* = 0.002) showed significant drops in abundance during the intervention but increased again after the return to individual diets (*p* = 0.061 and *p* = 0.001, respectively). Both serotonin (*p* = 0.026) and 5-hydroxytryptophan (*p* = 0.016) showed a significant increase during the intervention, while 5-hydroxytryptophan showed a significant drop afterwards (*p* = 0.013). Homoarginine was clearly elevated during the intervention (*p* = 0.006). Gamma-aminobutyric acid (GABA) was slightly increased during the intervention with a significant reduction for the subsequent period (*p* = 0.034). Uric acid was drastically elevated in the subsequent period after the intervention (*p* < 0.001), as was stearoylcarnitine (*p* = 0.014) (Figure 2).

### 3.6. Microbiota Assessment

The microbiome data had a different distribution than metabolomics data; hence, it was not possible to integrate them together.

### 3.7. Alpha and Beta Diversity

The alpha diversity at the ASV level did not differ between time points (*p* = 0.12, *p* = 0.54, and *p* = 0.30, respectively, for observed number, Shannon–Weiner index, and phylogenetic diversity), sex (*p* = 0.61, *p* = 0.14, and *p* = 0.54), or between those who used or did not use metformin (*p* = 0.79, *p* = 0.55, and *p* = 0.65), probiotics (*p* = 0.084, *p* = 0.06, and *p* = 0.058), or antibiotics (*p* = 0.46, *p* = 0.84, and *p* = 0.45). There were no correlations between alpha diversity at the ASV level and gastrointestinal symptoms, blood markers of glucose and lipid metabolism, leucocytes, liver enzymes, adipokines, SCFA, weight, blood pressure, or fecal calprotectin and zonulin levels. Alpha diversity at the genus level measured by Shannon differed regarding time point (*p* = 0.018), but not regarding sex (*p* = 0.410) or metformin (*p* = 0.950).

**Figure 3:**
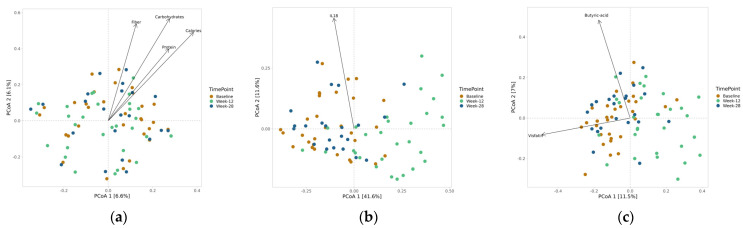
Significant correlations between beta diversity at ASV levels according to Bray–Curtis and breakfast contents (gram) (**a**) and according to weighted UniFrac and IL-18 (**b**). Significant correlations between beta diversity at genus levels according to Bray–Curtis and butyric acid and visfatin (**c**).

The only time point differences in beta diversity regarding ASV were found in weighted UniFranc, where a difference at 12 weeks was found compared with baseline and 28 weeks (*p* < 0.001). Differences were also found regarding metformin use (*p* < 0.01) but not antibiotic use (*p* = 0.058). Beta diversity at the ASV level based on Bray–Curtis differed regarding sex (*p* < 0.001), metformin use (*p* < 0.001), probiotics (*p* < 0.001), and antibiotics (*p* < 0.01). When correlating beta diversity at the ASV level with continuous variables, there was a correlation between breakfast intake in grams of carbohydrates (*p* = 0.003), fiber (*p* = 0.012), protein (*p* = 0.046), and calories (*p* = 0.006) and diversity according to Bray–Curtis, and a negative correlation between IL-18 (*p* = 0.045) and diversity according to weighted UniFranc (Figure 3a,b). Beta diversity based on Bray–Curtis at the genus level differed with time points (*p* < 0.001) and use of metformin and antibiotics (*p* < 0.001) and correlated with the concentration of butyric acid and visfatin (Figure 3c).

### 3.8. Taxonomy Measures

#### 3.8.1. Phylum

**Figure 4:**
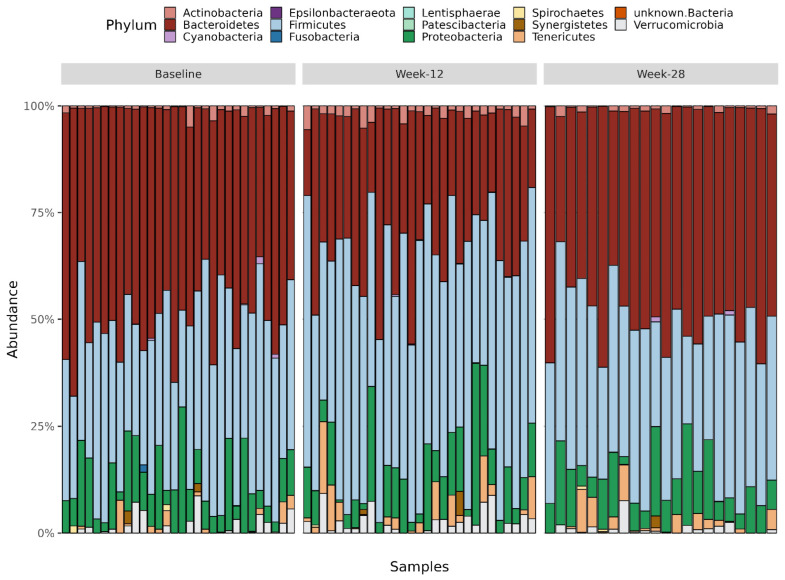
Abundance of different phyla at baseline, week-12, and week-28.

Fourteen different phyla were identified, i.e., Actinobacteria, Bacteroidetes, Cyanobacteria, Epsilonbacteraeota, Firmicutes, Fusobacteria, Lentispheaerae, Patescibacteria, Proteobacteria, Spirochaetes, Syneristetes, Tenericutes, unknown Bacteria, and Verrucomicrobia, with a clear dominance of Bacteroidetes and Firmicutes (Figure 4).

**Figure 5:**
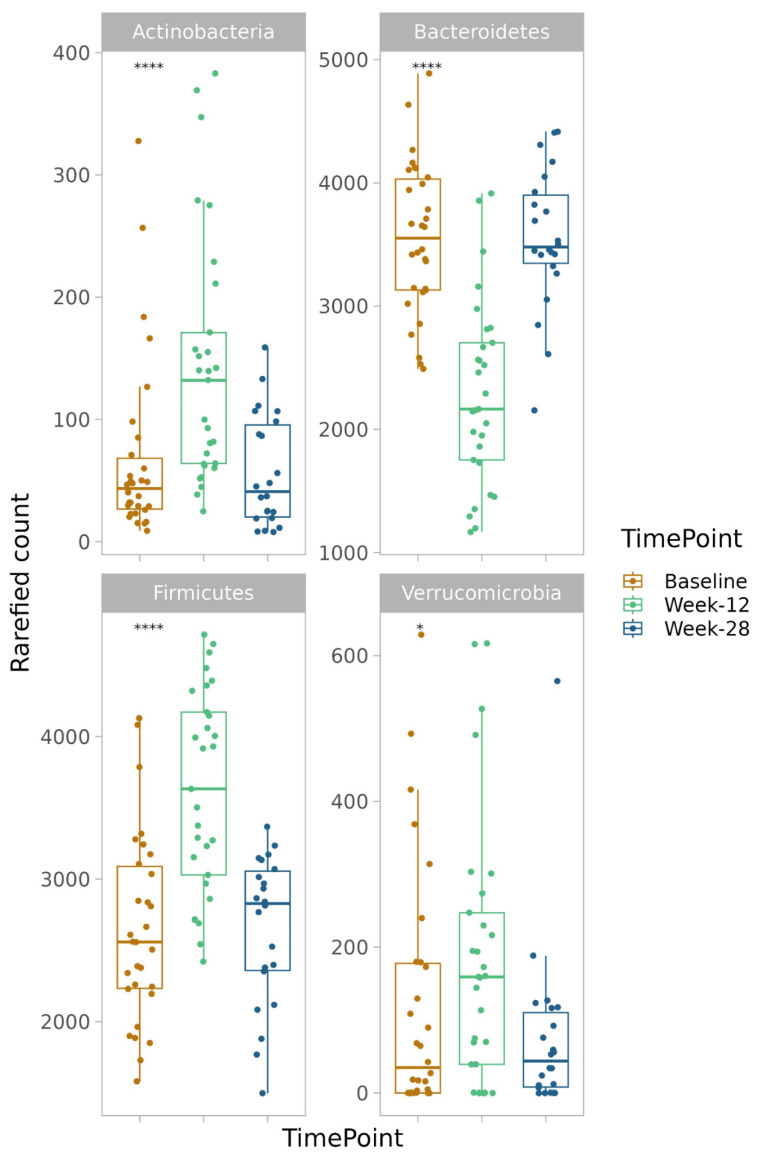
Significant differences in phylum abundance between baseline, week-12, and week-28. Kruskal–Wallis test. Patescibacteria was excluded in the figure due to very low abundance. **** = *p* < 0.0001, * = *p* < 0.05. *p* < 0.05 was considered statistically significant.

There was an increased abundance of Actinobacteria (*p* = 5.2 × 10^−5^), Firmicutes (*p* = 1.9 × 10^−6^), Patescibacteria (*p* = 0.04), and Verrucomicrobia (*p* = 0.049) after the intervention, whereas the abundance of Bacteroidetes decreased (*p* = 2.2 × 10^−8^) (Figure 5).

Correlations were found between Actinobacteria, Bacteroidetes, Firmicutes, and Verrucomicrobia; food components; and continuous variables of anthropometry, glucose and lipid homeostasis, and inflammation (Table 2).

#### 3.8.2. Genus

**Figure 6:**
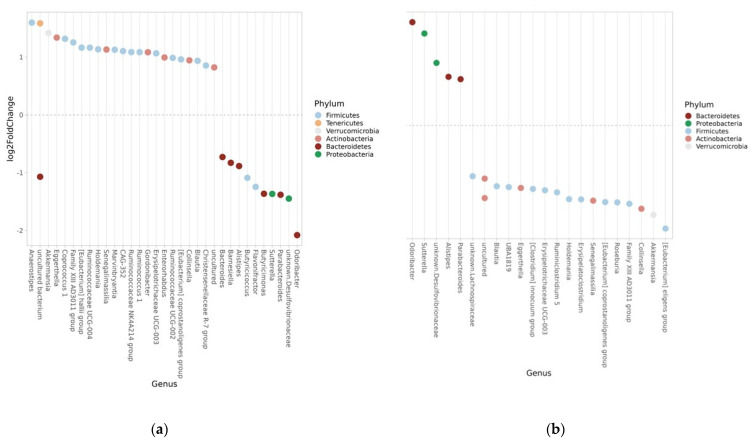
(**a**) Significant genera at week-12 compared to baseline. The microbes above the 0 line on the *y* axis are more abundant at week-12 and the ones below the 0 line on the *y* axis are more abundant at baseline. (**b**) Significant genera at week-28 compared to week-12. The microbes above the 0 line on the *y* axis are more abundant at week-28 and the ones below the 0 line on the *y* axis are more abundant at week-12.

There were several differences in abundance at the genus level between week 12, baseline, and week 28 (Figure 6a,b), where only the genus *Flavonitractor* (Firmicutes) decreased after 28 weeks compared to baseline (FDR = 0.018).

The greatest differences at week 12 compared to baseline were observed for *Anaerostipes* (Firmicutes)*, Family XIII AD3011* (Firmicutes), and *Odoribacter* (Bacteroidetes) (Table 3), and greatest differences compared to week 28 were observed for *Odoribacter*, *[Eubacterium] eligens* (Firmicutes), and *Sutterella* (Proteobacteria) (Table 4).

#### 3.8.3. ASV

**Figure 7:**
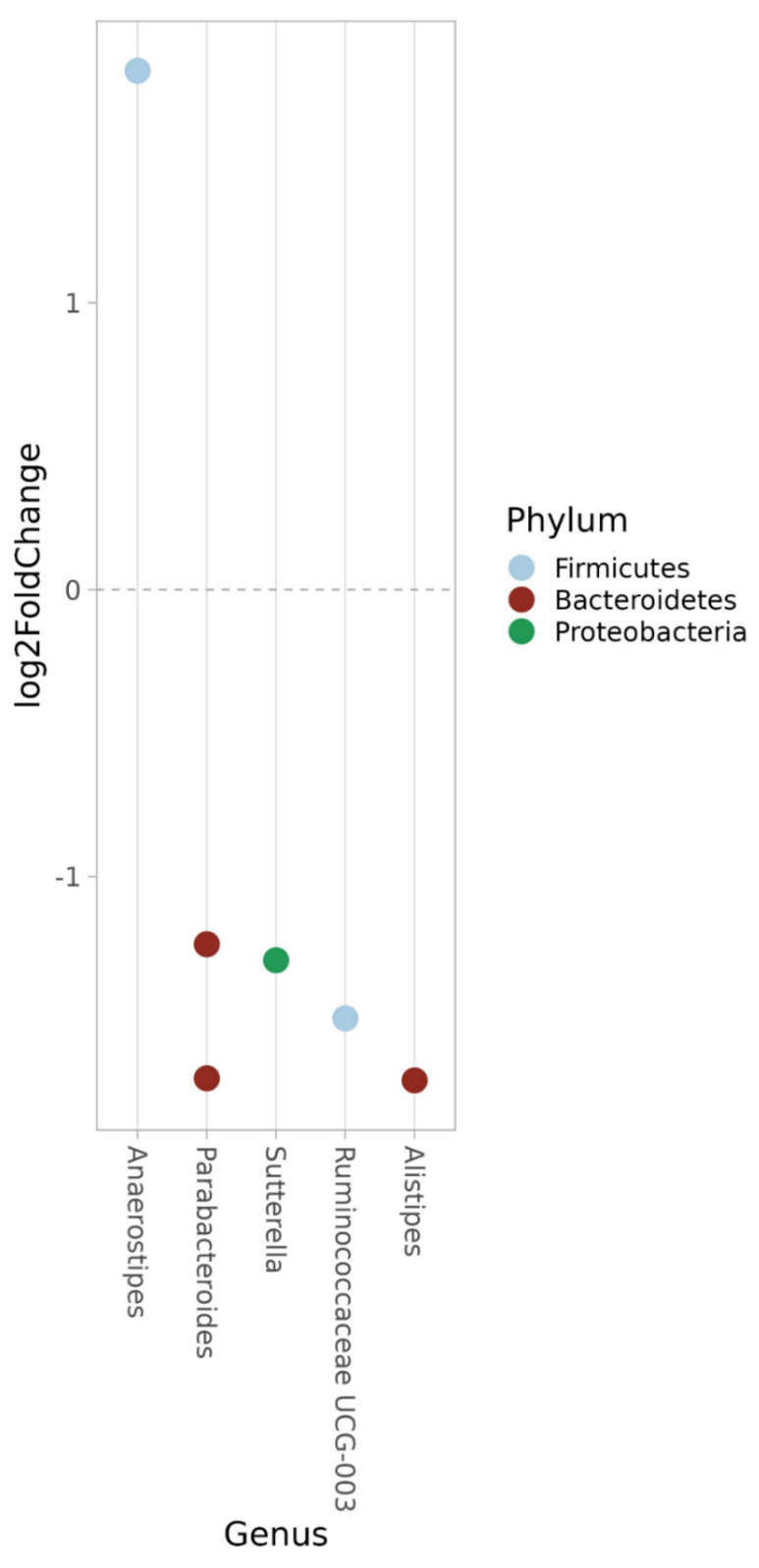
Significant amplicon sequence variants (ASV) at week-12 compared to baseline. The microbes above the 0 line on the *y* axis are more abundant at week-12 and the ones below the 0 line on the *y* axis are more abundant at baseline.

A few differences in abundance were observed at the ASV level between week 12 and baseline (Figure 7), whereas only an increase in an ASV belonging to the genus *Alistipes* (Bacteroidetes) (*p* = 1.93 × 10^−5^) was observed after week 28. No differences were observed between baseline and week 28 (EBI-ENA; project accession number: PRJEB63608).

## 4. Discussion

The intervention showed health benefits by clear reductions in BMI/weight and waist circumference, commonly used to estimate a healthy weight, but also showed metabolic alterations linked to specific food sources, reduced energy intake, and improved well-being. There were correlations between changes in food intake, microbiota diversity and abundance, anthropometry, markers of glucose and lipid metabolism, and inflammation. The changes in plasma metabolomics and gut microbiota were reversible as soon as the intervention was finished.

One of the important features of O-BN is reduced sucrose content. Sucrose-rich diets are responsible for deteriorated glucose parameters, increased weight, insulin resistance, and development of metabolic syndrome, as shown in animal studies [38]. In a mice model, sucrose affected the microbiota composition and delayed the recovery in antibiotic-associated diarrhea [39]. Regarding sugar consumption, fructose-containing sugars are especially associated with harmful effects in many health outcomes [40]. All dietary strategies to modify carbohydrate and fiber content in diabetes and metabolic syndromes showed gut microbiota changes, but with inconsistent changes without relation to clinical outcomes [6,11]. The most obvious finding was a decrease in diastolic blood pressure and triglycerides [11], which was also found after O-BN [13]. O-BN decreased levels of glucose, HbA1c, and insulin and reduced the insulin resistance [13] in contrast to many other diets which only decreased the HbA1c levels [2,3,6]. Overall, high-fiber, whole-diet interventions are more beneficial than other interventions, independent of carbohydrate intake, and are more beneficial than fiber supplements [2,3,6].

The metabolic changes observed during the intervention may mediate several of the clinical effects observed, in alignment with hormonal changes [41]. The compound 1,5-AG is a validated marker of short-term glycemic control [42]. This compound was increased after 12 and 28 weeks compared to baseline, in linkage with improved glycemic control and lower levels of glucose and insulin during the study [13]. Also, the drop in thyroxine may be coupled to lower glucose levels due to its modulating effect on hepatic insulin sensitivity and metabolism [43]. The drastic drop of chiro-inositol levels during the subsequent period might have had a negative effect in the aspect of insulin resistance since this metabolite has been linked to beneficial effects in diabetes treatment [44]. At baseline, the participants had higher levels of cholesterol and triglycerides, which were decreased after 12 weeks. Accordingly, most of the lyso-lipids showed strong significant reductions during the intervention and an overall strong significant increase for the subsequent post-intervention period in alignment with the fluctuations in lipid concentrations [13,45]. Lyso-lipids are commonly associated with inflammation and immune responses [46], which further support possible negative health consequences when leaving the dietary intervention.

Accumulated levels of cycloleucine in skeletal muscle have historically been associated with starvation [47]. The largest change in the present study was the increased abundance of circulating cycloleucine, supporting reduced energy intake during the intervention period.

Cortisol and cortisone levels, linked to stress responses [48], dropped during the intervention followed by an increase afterwards, indicating negative health effects when returning to diets resulting in increased blood glucose and insulin levels [49]. Serotonin and 5-hydroxytryptophan are linked to disorders such as depression and associated with happiness and hunger sensations [50]. The increased levels of these hormones during the intervention may explain the reported increase in satiety, mental health, and vitality [13,17]. High levels of homoarginine have been coupled with improved cardiovascular health [51], whereas a loss of GABA has been associated with diabetes pathogenesis [52], suggesting that the dietary changes may also prevent secondary disease complications. The increased levels of uric acid indicate possible hyperuricemia. Hence, by returning to their previous diet—and possibly drastically increasing the intake of fast-acting carbohydrates—it can lead to hyperuricemia, hyperinsulinemia, and impairment/development of T2D [53]. Increased intake of wheat/glutinous fast-acting carbohydrates after the intervention was reflected by elevated levels of stearoylcarnitine, which has been associated with celiac disease [54].

In a systematic review regarding gut microbiota and T2D, a less healthy diet with increased carbohydrate, fat, or energy content, or a reduced fiber intake, was associated with diabetes, and the dietary intake caused significant variations in bacterial abundance [55]. Differences in abundance of bacteria in the current study were found at 12 weeks. Thus, the secondary changes in microbiota induced by dietary changes are reversible as soon as subjects go back to their ordinary food habits.

The changes of beta diversity following the intervention were in line with previous dietary interventions of adult T2D [6]. There was increased abundance of *Akkermansia*, but not *Faecalibacterium prausnitzii*, microbes assumed to be involved in the development of T2D [56]. The abundance of *Alistipes* was higher at follow-up, in line with the finding that *Alistipes* has been found in increased abundance among T2D patients [55]. This bacterium has protective effects against certain diseases including cardiovascular diseases, whereas it may be pathogenic in others due to its inflammatory potential [57].

The O-BN lowered the abundance of Bacteroidetes, and elevated the abundance of Actinobacteria and Firmicutes, in accordance with dietary interventions of whole-grain fiber supplements in healthy subjects [5]. No effect on the abundance of *Bifidobacterium* was found, in line with some studies [6], although it was affected in some others [3,4]. The importance of whole-grain cereal foods is not only to increase fiber intake but also to increase the intake of vitamins, minerals, and antioxidants found in the bran layers. Many low carbohydrate diets, e.g., low FODMAP, reduce the content of fiber, including resistant starch important for maintenance of normoglycemia [38], which may counteract other healthy effects of the diet [2]. The advantage of O-BN compared with many other low-carbohydrate diets is maintaining high intake of fiber and whole grains [4].

Diabetes is associated with low-grade inflammation [2]. Some diets are considered inflammatory [58]. However, dietary interventions in diabetes have been shown to have minimal or no effect on inflammation, SCFA, and anthropometry in several studies [3,5,6,59]. On the other hand, correlations between diet and microbiota have been established [3,4,5], as well as between inflammation and gut microbiota [2]. The current inverse correlation between beta diversity, Firmicutes abundance, and IL-18 is interesting. IL-18 produced in the enteric nervous system plays a key role in intestinal homeostasis and host defense against intestinal infections [60]. Absolute values of IL-18 correlated with the circulating levels of glucose, HbA1c, cholesterol, and triglycerides at the different time points [61]. Breakfast intake was the only difference in food intake among subjects and, therefore, was used for calculations of correlations. Gut microbiota changes correlated with changes of breakfast content and IL-18, and changes in breakfast intake of carbohydrates and fiber tended to correlate with IL-18. In accordance with our findings of correlations between food, gut microbiota, and clinical outcomes, some probiotics and fiber supplemental studies showed correlations between bacteria, food components, metabolic profile, and inflammatory markers, which were not found in many other dietary interventions [2,6,11,55]. The question remains whether these correlations reflect causality. Nevertheless, inverse correlation between changes of IL-18 and beta diversity during the O-BN reflects a lower degree of inflammation and an improved glycemic and lipid metabolism [13,61]. Other mechanisms than microbiota changes may be involved in symptom development [62].

The strength of the study is that all food except breakfast was delivered to the participants. Thus, all had mainly the same food intake. One of the limitations is the small sample size. Individuals being their own controls may be both a weakness and strength. *Bifidobacterium* is hard to investigate in V1–V2 16S genes, which may have influenced the absence of differences between time points. Analyzing microbiomes with whole metagenomes could have also given us a better understanding of the functional capabilities of these microbes. However, the challenge of linking them to the differences we see in metabolomics still remain. This is due to the fact that changes in other factors like hormones and transmitters have a higher influence on the circulating metabolites [41]. Heterogeneity in study design, dietary composition, and study cohorts explains the divergent results among studies.

## 5. Conclusions

In conclusion, O-BN induced changes in metabolomics and gut microbiota which correspond to the previously reported clinical outcomes of weight reduction, improved glucose homeostasis and lipid metabolism, and reduced IL-18 levels. This underlines the tight correlations between food, gut microbiota, metabolism, and inflammation. The reversibility of changes from 12 to 28 weeks indicates the importance of continuous healthy lifestyle habits, and not only temporary solutions.

## Figures and Tables

**Table 1:** Patient characteristics at inclusion.

Gender (F/M)	17/13	Antihypertensive Medication (%)	63
Age (years)	57.5 ± 8.2	Lipid-lowering medication (%)	47
BMI (kg/m^2^)	29.9 ± 4.1	IBS (%)	13
Education (%)		Diabetes duration (years)	10.4 ± 7.6
Primary school	16		
High school	57	Diabetes management (%)	
University	27	Diet alone	7
Occupation (%)		Metformin	40
Employed	67	Sulfonylurea	3
Retired	17	Sulfonylurea and metformin	7
Sick leave	13	DPP-4 inhibitor and metformin	3
Unemployed	3	Metformin and insulin	27
Smokers (%)	23	Insulin	13
Snuff users (%)	23	Diabetes complication (%)	
Frequency of alcohol intake (%)		Retinopathy	27
None	10	Nephropathy	17
Once a month or less	50	Neuropathy	30
2–4 times a month	27	Gastroparesis	3
2–3 times a week	13	Macroangiopathy	17
Physical activity (%)		Antibiotic use last 6 months	
Sedentary leisure time	7	During intervention	20
Moderate exercise during leisure	53	During follow-up	35
Moderate regular exercise during leisure	27	Probiotic use (*Lactobacuillus plantarum* DSM 9843)	17
Regular exercise and training	13		

BMI = Body Mass Index, IBS = Irritable Bowel Syndrome, HbA1c = Glycosylated Hemoglobin, DPP-4 = Dipeptidyl Peptidase-4. Results of age, BMI, total energy intake, diabetes duration, and HbA1c values are given as means ± standard deviation (SD) and other information in the percentage of participants (%), *n* = 30.

**Table 2:** Correlations between phyla and continuous variables of metabolic characteristics.

Variables	Actinobacteria	Bacteroidetes	Firmicutes	Verrucomicrobia
***Anthropometry***				
Waist circumference	R^2^ = 0.05, *p* = 0.038			R^2^ = 0.08, *p* = 0.013
Weight				R^2^ = 0.07, *p* = 0.016
Systolic blood pressure		R^2^ = 0.08, *p* = 0.01	R^2^=−0.13, *p* = 0.001	
Diastolic blood pressure		R^2^ = 0.14, *p* = 8 × 10^−4^	R^2^=−0.08, *p* = 0.014	
***Inflammatory markers***				
CRP	R^2^ = 0.08, *p* = 0.011			
IL-18			R^2^=−0.14, *p* = 0.009	
***HbA1c***		R^2^ = 0.08, *p* = 0.013	R^2^=−0.12, *p* = 0.002	R^2^ = 0.06, *p* = 0.034
***Lipids***				
Cholesterol		R^2^ = 0.09, *p* = 0.005		
Triglycerides			R^2^=−0.05, *p* = 0.039	R^2^ = 0.05, *p* = 0.037
***Short-chain fatty acids***				
Butyric acid	R^2^ = 0.07, *p* = 0.02	R^2^=−0.10, *p* = 0.004		R^2^ = 0.06, *p* = 0.022
Isobutyric acid		R^2^=−0.05, *p* = 0.037		
***Zonulin feces***	R^2^ = 0.13, *p* = 0.001	R^2^ = 0.07, *p* = 0.013		
***Breakfast intake***				
Carbohydrate E%			R^2^ = 0.18, *p* = 0.028	
Protein E%		R^2^=−0.20, *p* = 5 × 10^4^	R^2^ = 0.10, *p* = 0.019	R^2^ = 0.08, *p* = 0.035

CRP = C-reactive protein, E% = energy percent, HbA1c = hemoglobin A1c. Correlations were calculated by the ‘envfit’ function in the ‘vegan’ package of R over the whole study period. *p* < 0.05 was considered statistically significant.

**Table 3:** Differences in genus levels at week 12 versus baseline.

Phylum	Genus	Log2-Fold Change	FDR
Firmicutes	Family XIII AD3011	1.254	<0.001
Firmicutes	Anaerostipes	1.596	0.001
Firmicutes	Ruminococcaceae UCG-004	1.163	0.008
Firmicutes	Erysipelotrichaceae UCG-003	1.065	0.009
Firmicutes	Blautia	0.936	0.010
Firmicutes	Ruminococcaceae NK4A214	1.086	0.014
Firmicutes	[Eubacterium] coprostanoligenes	0.960	0.019
Firmicutes	Ruminococcaceae UCG-002	0.986	0.019
Firmicutes	Marvinbryantia	1.128	0.019
Firmicutes	Holdemania	1.135	0.019
Firmicutes	Coprococcus 1	1.316	0.019
Firmicutes	Butyricicoccus	−1.088	0.019
Firmicutes	Flavonifractor	−1.244	0.019
Firmicutes	CAG-352	1.103	0.031
Firmicutes	Ruminococcus 1	1.085	0.032
Firmicutes	Christensenellaceae R-7	0.854	0.034
Firmicutes	[Eubacterium] halli	1.163	0.036
Verrucomicrobia	Akkermansia	1.417	0.005
Actinobacteria	Eggerthella	1.337	0.004
Actinobacteria	Collinsella	0.944	0.009
Actinobacteria	Senegalimassilla	1.131	0.014
Actinobacteria	Gordonibacter	1.084	0.019
Actinobacteria	Enterorhabdus	0.993	0.032
Bacteroidetes	Barnesiella	−0.828	0.031
Bacteroidetes	Bacteroides	−0.728	<0.001
Bacteroidetes	Alistipes	−0.884	<0.001
Bacteroidetes	Butyricimonas	−1.363	<0.001
Bacteroidetes	Parabacteroides	−1.381	<0.001
Bacteroidetes	Odoribacter	−2.0.79	<0.001
Proteobacteria	Sutturella	−1.366	<0.001

Benjamini–Hochberg, or false discovery rate (FDR), was used to correct for multiple comparisons of abundances. FDR < 0.05 was considered statistically significant.

**Table 4:** Differences in genus levels at week 28 versus week 12.

Phylum	Genus	Log2-Fold Change	FDR
Bacteroidetes	Odoribacter	1.861	<0.001
Bacteroidetes	Alistipes	0.876	0.003
Bacteroidetes	Parabacteroides	0.833	0.005
Proteobacteria	Sutterella	1.657	<0.001
Proteobacteria	Unknown Desulfovibrionaceae	1.127	0.020
Firmicutes	Blautia	−1.096	0.007
Firmicutes	UBA1819	−1.111	0.044
Firmicutes	Family XIII AD3011	−1.412	0.002
Firmicutes	Erysipelatoclostridium	−1.333	0.030
Firmicutes	[Eubacterium] coprostanoligenes	−1.381	0.003
Firmicutes	Holdemania	−1.328	0.018
Firmicutes	[Clostridium]	−1.144	0.045
Firmicutes	Erysipelotrichaceae	−1.169	0.012
Firmicutes	[Eubacterium] eligens	−1.858	0.002
Firmicutes	Roseburia	−1.387	0.003
Firmicutes	Ruminiclostridium	−1.205	0.044
Firmicutes	Unknown Lachnospiraceae	−0.915	0.020
Actinobacteria	Eggerthella	−1.127	0.043
Actinobacteria	Collinsella	−1.501	<0.001
Actinobacteria	Senegalimassilia	−1.356	0.009
Actinobacteria	Akkermansia	−1.614	0.004

Benjamini–Hochberg, or false discovery rate (FDR), was used to correct for multiple comparisons of abundances. FDR < 0.05 was considered statistically significant.

## Data Availability

The clinical data that support the findings of this study are available on request from the corresponding author. The data are not publicly available due to privacy or ethical restrictions. The raw sequencing data of the 16rRNA amplicons from the patients involved in this study are available for download at EBI-ENA under the project accession number: PRJEB63608.

## Supplementary Materials

The following supporting information can be downloaded at: https://www.mdpi.com/article/10.3390/nu15143273/s1, Figure S1: Principal component analysis (PCA) of metabolomics of GC-MS (A), LC-MS (B), and Lipidomics (C); Table S1: Description of food items included in the Okinawa-based Nordic diet; Table S2: Nutrition composition and daily mean intake of energy, nutrients, and food components of the Okinawa-based Nordic diet; Table S3: Description of the breakfast composition in the subjects; Table S4: Amount of fat and fiber in the diet to type 2 diabetes patients before, during and after the 12-week interventional study; Table S5: Anthropometric and metabolic characteristics of the type 2 diabetes cohort; Table S6: Visual Analog Scale for Irritable Bowel Syndrome for evaluation of gastrointestinal symptoms; Nutrition Questionnaire; Study Protocol.
