# Supplementary material for: An Okinawan-Based Nordic Diet Leads to Profound Effects on Gut Microbiota and Plasma Metabolites Linked to Glucose and Lipid Metabolism
Source: Nutrients. 2023 Jul 24;15(14):3273. doi: 10.3390/nu15143273 (PMC10384944; doi:10.3390/nu15143273)
Supplement: Supplementary file 1 [file nutrients-15-03273-s001.zip › nutrients-2507364-supplementary.pdf]

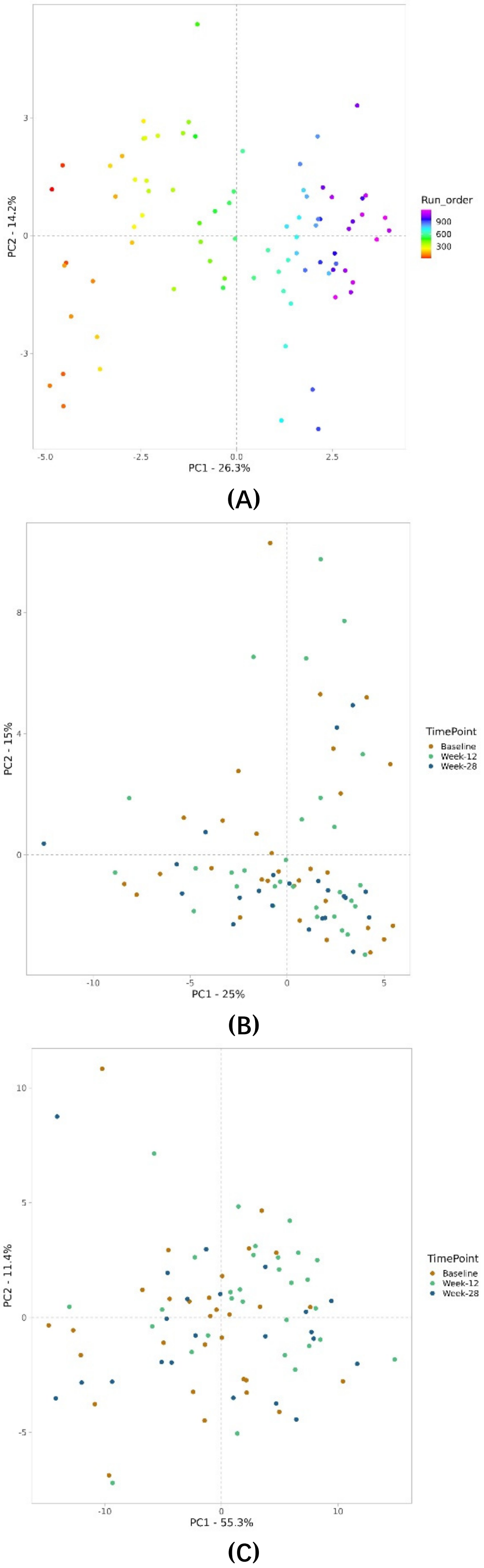

Figure S1. Principal component analysis (PCA) of metabolomics of GC-MS (A), LC-MS (B), and Lipidomics (C).

Supplementary Table S1. Description of food items included in the Okinawan-based Nordic diet

**Food groups**	
Root vegetables	Hot or cold. Red or yellow carrots as snacks or incorporated in the dish. Parsnips, parsley root, rutabaga, celery root, beetroot, artichoke, or sweet potatoes as part of the dishes.
Potatoes	Shredded in potatoes burger, fried raw.
Vegetables	Onion, leek, garlic, cabbage, cauliflower, broccoli, squash, eggplant, fennel, spinach, various types of salad, tomato, pepper, cucumber, mushroom, and asparagus.
Legumes	Fresh; green peas, sweet peas, and soybean (edamame).<br>Dried; white and black beans, kidney beans, and lentils.
Nuts	Walnuts, almonds, and cashews.
Seeds	Sunflower seeds, pumpkin seeds, linseed, and sesame seed, as part of snacks served with fruits and berries.
Fruits and berries	Apple, orange, pear, strawberry, blueberry, lingonberry, dried apricots, and prunes. Coco nut milk was used for cooking.
Meat products and poultry	Chicken, rooster, turkey, game meat (hart and deer), and ground beef.
Fish and seafood	Salmon, codfish, plaice, mackerel, herring, and prawns.
Egg	Eggs were used for cooking and as snacks.
Dairy products	Low-fat drinking milk (maximum 1.5% fat), filmjölk (a Swedish fermented product similar to yogurt) and low-fat yogurt (1.5% fat), hard cheese (17% fat), parmesan cheese, cottage cheese, and quark. Whipped cream (36% fat) and soy cream used for cooking, and Turkish yogurt (10% fat).
Fat and oil	Vegetable oil including rapeseed oil, sesame oil, and olive oil for cooking and dressing. Vegetable fat spread (60% fat) for bread.
Rice	Whole-grain rice and black Thai rice. A maximum of half a dl of uncooked rice per serving.
Cereals	Rolled oats, rye flakes, whole-grain whole-kernel rye, oat, barley, and wheat. Bread rich in whole-grain, a maximum of two slices per day. Whole-grain pasta, bean paste, millet, oat bran, quinoa, and rye flour.
Beverages/liquids (non-alcoholic)	Recommended free amount of tap water or mineral water with the meals. Light products to be avoided. Tea, filtered coffee, or instant coffee.
Spices	Own preference with restrictions on salt.
Sweets and desserts	Homemade dark chocolate (>70%), bean truffles, coconut flakes, ginger, and prune cake. No refined sugar or sweetener was used, but instead natural sweet foods like prunes, pears, and occasionally honey.
Alcoholic beverages	Maximal intake of alcoholic beverages was set to 30 g ethanol/week.

Supplementary Table S2. Nutrition composition and daily mean intake of energy, nutrients, and food components of the Okinawa-based Nordic diet

Nutritional value	Unit	Calculated value	E%	Recommended (NNR 2012)
Total Energy	kcal	1866		
Energy (excluding beverages)	kcal	1629		
Protein	g	95.0	23 E%	10–20 E%
Fat	g	63.9	35 E%	25–40 E%
Saturated fatty acids	g	18.7	10 E%	<10 E%
Polyunsaturated fatty acids	g	14.9	8 E%	5–10 E%
Mono-unsaturated fatty acids	g	17.8	10 E%	10–20 E%
Carbohydrates	g	168.4	42 E%	45–60 E%
Sucrose	g	23.5	6 E%	<10 E%
Dietary fiber	g	35.9	4 E%	25–35 g
alpha-Tocopherol	mg	1.9		
beta-Carotene	µg	9902.1		
Retinol	µg	259.7		
Vitamin A	µg	139.9		700
Vitamin D	µg	8.8		10
Vitamin E	mg	11.4		8
Thiamine	mg	1.1		1.1
Riboflavin	mg	1.2		1.2
Niacin equivalent	mg	34.5		14
Niacin	mg	19.7		14
Vitamin B6	mg	2.1		1.2
Folate	µg	386.1		300
Vitamin B12	µg	10.4		2
Vitamin C	mg	303.0		75
Sodium	mg	2401.1		2300
Potassium	mg	3385.6		3100
Phosphorus	mg	1446.7		600
Calcium	mg	840.4		800
Iron	mg	10.7		15
Magnesium	mg	317.4		280
Zinc	mg	9.7		7
Iodine	µg	34.9		150
Selenium	µg	61.0		50

(n=30). The results are calculated on the intake of a normal week.The daily average calorie content of the meals are given in kcal and the energy percentage (E%) is given for protein, fat, saturated fatty acids, polyunsaturated fatty acids, mono-unsaturated fatty acids, carbohydrate, and sucrose, respectively. Recommendations for a traditional diet according to the Nordic Nutrition Recommendations are shown for comparison [14]. Recommendations for total daily energy intake are not given since they are individual and differ between subjects. Vitamins A and D, and Iodine may be underestimated due to lack of data.

Supplementary Table S3. Description of the breakfast composition in the subjects

Subjects	Breakfast at baseline					Breakfast at week 12				
	Energy **(kcal)**	***Protein*** **(gram/ E%)**	***Fat*** **(gram / E%)**	***Carb*** **(gram / E%)**	***Fiber*** **(gram / E%)**	***Energy*** **(kcal)**	***Protein*** **(gram / E%)**	***Fat*** **(gram / E%)**	***Carb*** **(gram / E%)**	***Fiber*** **(gram / E%)**
1	488	22/18	33/59	25/20	6/2	331	17/21	17/46	24/30	7/4
2	184	7/15	3/15	29/64	6/7	258	13/20	10/33	27/42	7/5
3	301	12/16	22/63	14/19	3/2	267	16/24	15/50	15/23	4/3
4	191	10/20	12/55	11/24	0,7/0,7	420	28/28	16/34	37/36	5/2
5	209	8/16	5/21	31/60	5/5	248	10/17	8/28	30/49	8/6
6	241	8/14	5/17	38/64	8/6	81	3/15	2/26	10/52	3/8
7	515	20/16	21/36	55/43	12/5	362	24/27	13/33	33/37	5/3
8	230	10/18	11/40	22/39	3/2	537	33/25	19/32	53/40	8/3
9	526	26/20	24/40	49/38	5/2	309	23/30	10/29	31/41	5/3
10	260	9/14	12/41	28/43	3/2	248	14/23	9/30	26/42	5/5
11	119	7/24	6/41	9/30	3/5	131	8/25	7/44	8/26	3/5
12	298	10/13	9/26	43/58	5/3					
13	328	16/19	3/9	56/70	5/3	310	13/17	10/29	39/51	5/3
14	443	18/16	20/41	45/41	5/2	141	8/22	6/40	12/35	3/3
15	332	11/14	8/22	51/62	4/2	221	16/30	5/21	25/46	4/4
16	213	7/13	3/13	36/69	5/5	253	15/24	9/32	24/39	6/5
17	291	10/14	14/43	28/40	8/5	537	15/12	33/55	40/31	9/3
18	497	21/17	27/48	41/34	4/1	188	11/24	4/19	14/30	4/4
19	300	12/16	13/38	31/43	10/6	385	18/19	15/35	42/44	8/4
20	343	13/16	16/40	33/40	8/4	175	9/21	9/44	14/32	4/4
21	383	15/16	11/26	51/54	8/4	299	10/13	6/17	49/67	8/5
22	590	25/17	29/44	55/38	4/1					
23	390	17/17	11/26	51/53	8,5/4	364	14/16	15/37	38/42	9/5
24	555	19/14	26/42	56/41	11/4	488	25/21	15/27	58/48	9/3
25	502	25/20	18/32	57/46	5/2	341	19/23	17/43	27/32	5/3
26	212	10/19	4/17	31/59	6/5	265	15/23	3/11	39/60	9/6
27	465	20/18	18/32	53/46	4/2	355	24/27	14/35	31/36	5/3
28	290	15/21	17/53	18/25	2/1					
29	153	6/17	3/19	24/65	4/4	305	15/20	9/27	38/51	8/5
30	454	21/19	16/31	52/47	7/3	374	20/21	11/26	45/50	7/3

(N = 30). Carb = carbohydrates, E% = energy percentage

Supplementary Table S4. Amount of fat and fiber in the diet to type 2 diabetes patients before, during and after the 12-week interventional study

**Subjects**	**Fat**			**Fiber**		
	Baseline	***Week 12***	***Week 28***	***Baseline***	***Week 12***	***Week 28***
1	38	5	10	11	14	12
2	42	15	35	17	16	15
3	31	22	23	11	14	11
4	61	51	34	8	17	16
5	43	13	12	12	16	15
6	43	20	31	14	15	14
7	50	14	21	13	13	12
8	56	22	34	10	15	13
9	87	29	20	13	13	8
10	75	22		9	12	
11	33	34	21	10	11	12
12	51			9		
13	19	4		13	13	
14	73	48	36	8	11	10
15	28	12	23	9	14	10
16	28	31	11	11	15	13
17	45	38	55	12	13	13
18	50	23	16	14	16	16
19	33	26	36	15	16	16
20	68	14	39	13	16	14
21	60	42		13	12	
22	47			10		
23	39	11	28	13	12	13
24	40	2	25	10	16	14
25	40	7	33	22	15	16
26	23	3	3	13	16	16
27	41	33	33	14	16	16
28	60	22		13	14	
29	27	5		12	14	
30	23	21	22	14	16	14

The scores are calculated from the nutrition questionnaire, where the scores for fat means: <40 is rather small amounts of fat, 40-50 is good, 51-71 is too much fat and > 71 is quite too much. The scores for fiber means: 17-20 is very good, 12-16 is rather good, may be improved, and 6-11 is too low [18].

Supplementary Table S5. Anthropometric and metabolic characteristics of the type 2 diabetes cohort

Variable	Mean ± SD	P-value
Weight (kg)		
Baseline	89.8 ± 15.0	
Week 12	83.5 ± 14.3	<0.001
Week 28	83.6 ± 15.2	<0.001
BMI (kg/m^2)		
Baseline	29.9 ± 4.1	
Week 12	27.9 ± 4.1	<0.001
Week 28	28.4 ± 4.6	<0.001
Waist circumference		
Baseline	107.3 ± 11.0	
Week 12	101.0 ± 11.5	<0.001
Week 28	99.3 ± 11.2	<0.001
Systolic blood pressure		
Baseline	140.2 ± 16.3	
Week 12	1130.4 ± 14.5	<0.001
Week 28	137.8 ± 12.7	0.643
Diastolic blood pressure		
Baseline	82.3 ± 11.9	
Week 12	74.8 ± 10.3	0.001
Week 28	77.6 ± 8.8	0.060
Fasting glucose (mmol/L)		
Baseline	9.7 ± 3.5	
Week 12	7.9 ± 2.7	<0.001
Week 28	9.0 ± 3.3	0.556
HbA1c (mmol/mol)		
Baseline	62.9 ± 18.7	
Week 12	49.1 ± 10.3	<0.001
Week 28	53.1 ± 13.3	0.010
Insulin (mIU/L)		
Baseline	15.5 ± 9.7	
Week 12	11.7 ± 6.7	<0.001
Week 28	12.8 ± 7.4	0.064
Triglycerides (mmol/L)		
Baseline	1.8 ± 1.1	
Week 12	1.5 ± 0.9	0.018
Week 28	1.9 ± 1.6	0.250
Cholesterol (mmol/L)		
Baseline	4.6 ± 0.8	
Week 12	4.2 ± 1.0	0.003
Week 28	4.6 ± 0.8	0.875
Interleukon-18		
Baseline	226 ± 26	
Week 12	189 ± 26	0.002
Week 28	201 ± 27	0.212
Fat intake (score)		
Baseline	45.1 ± 16.7	
Week 12	21.0 ± 13.5	<0.001
Week 28	26.1 ± 11.6	<0.001
Fiber intake (score)		
Baseline	12.2 ± 2.8	
Week 12	14.3 ± 1.7	0.001
Week 28	13.0 ± 3.4	0.416

N = 30 subjects. The mean values and standard deviations (SD) are presented for body mass index (BMI), fasting blood values of hemoglobin A1c (HbA1c); fasting serum levels of insulin; and fasting plasma values of glucose, triglycerides, and cholesterol. Analyses are performed by standard methods at the Department of Clinical Chemistry.

Values are shown at inclusion (baseline), 12 weeks after diet intervention and 16 weeks after the end of diet intervention (week 28, N = 23). Comparisons were made between each time point and baseline, using Student´s paired test. P-value < 0.05 was considered statistically significant.

Supplementary Table S6. Visual Analog Scale for Irritable Bowel Syndrome for evaluation of gastrointestinal symptoms

**Variable**	**Median (interquartile range)** **N = 30**	**P-value**
Pain		
Baseline	6 (2-22)	
Week 12	3 (2-15)	0.033
Week 28	3 (2-7)	0.192
Diarrhea		
Baseline	8 (3-35)	
Week 12	3 (2-16)	0.023
3Week 28	2 (1-6)	0.020
Constipation		
Baseline	4 (1-21)	
Week 12	3 (2-24)	0.806
Week 28	4 (2-20)	0.703
Bloating		
Baseline	22 (5-55)	
Week 12	8 (2-35)	0.085
Week 28	6 (2-20)	0.015
Nausea		
Baseline	3 (1-8)	
Week 12	2 (1-4)	0.466
Week 28	2 (2-4)	0.112
Psychological well-being		
Baseline	19 (4-38)	
Week 12	5 (2-16)	0.002
Week 28	4 (2-15	0.049
Influence on daily life		
Baseline	6 (1-35)	
Week 12	4 (2-28)	0.522
Week 28	3 (1-9)	0.558

Symptoms assessed on a VAS scale 0-100 where 0 means absence of symptoms and 100 means maximal symptoms [19]. The median and interquartile mean values at inclusion (baseline), 12 weeks after diet intervention, and 16 weeks after the end of diet intervention (week 28). Increase of values indicate improved symptoms. Comparisons were made between each time point and baseline, using Wilcoxon´s test. P-value <0.05 is considered statistically significant.

19 questions about your food habits

Mark the letter after the question which best matches your eating habits.

Fat Fiber

1. On my sandwiches, I usually spread

A butter, Bregott or margarine (80 %)……………….………….2

B light margarine (e.g. Lätta, Becel, medium Bregott) ...…….1

C no fat…….……………………………………..................0

2. As topping I most often have

A cheese (28%), sausage, paté………………………………3

B cheese (17%), lean ham, hamburger meat, mackerel ....….1

C marmelade, honey……………………………….….…0

D no topping………………………………………………...0

E A as often as B……………………………………………2

3. I eat bread (even hard bread) every day

A maximal 2 slices..…………………………………………1

B 2-5 slices………………………………………………….2

C 6-9 slices………………………………………………….4

D minimal 10 slices……………………………………….5

4. I mostly eat

A white bread……………………………………………….1

B loaf, bread………………………………………………...1

C coarse rye bread, crisp bread, wholemeal bread (no visible grains)...2

D whole grains (partly with whole grains)…………………….3

5. Of milk, fil and youghurt I drink/eat

A At least 1 liter daily………………………………………6

B 3-10 dl daily……………………………………………….4

C At most .2dl daily.………………………………………1

D Almost nothing……………………………………………..0

6. Of milk, fil and yoghurt I drink/eat

A most standard milk products (3% fat)……………………………6

B most medium milk products (1.5% fat)…………………….......3 → ☐ X ☐ = ☐

C most skimmed milk products (0.5% fat)……………………....1

☐ ☐

Sum to next page:

	Fat	Fiber
Sum from previous page:	☐	☐

7. I eat porridge, muesli or breakfast cereals

A seldom or never……………………………………………..1

B some times weekly…..…………………………………….2

C almost daily……………………………………………..3

→ Fiber ☐

8. For lunch or dinner I eat all meat, mince or sausage (except venison or chicken)

A almost daily………..……………………………………20

B some times weekly…..…………………………………10

C once weekly…...…………………………………………3

D more seldom or never……………………………………1

→ Fat ☐

9. Fruit or berries I eat

A seldom..……………………………………………………1

B some times weekly………………………………………2

C daily…..……………………………………………………3

→ Fiber ☐

10. Whipped cream or crème fraiche, fat (also in sauces and the like) I eat

A a few times weekly or more seldom.…………………10

B about once weekly……..………………………………3

C more seldom or never…………………………………1

→ Fat ☐

11. I eat chips or other types of snacks

A almost daily………..…………………………………..20

B some times weekly……………………………………10

C once daily……..……………………………………..3

D more seldom or never..…………………………………1

→ Fat ☐

12. I eat chocolate pieces and sweets

A almost daily………. ………………………………….30
B some times weekly ……………………………………15
C once daily ……..……………………………………..4
D more seldom or never …………………………………..1

13. I eat coffee bread or ice cream

A almost daily ……………………………………..……..12
B some times weekly ……………………………………..6
C once daily ……..………………………………………..2
D more seldom or never …………………………………..1

14. Vegetables

(except tomatoes, cucumber or lettuce) I eat

A a few times weekly or more seldom ..………………..1
B once weekly …..……………………………………..2
C more than once daily…..……………………………..3

15. I eat French fries or fried potatoes

A some times weekly……………………………….22
B once weekly…… ………………………………….6
C a few times monthly…………………………………3
D more seldom or never ……………………………….1

16. I eat root vegetables and other types of vegetables

A. some times weekly, or more seldom…………………1
B. once daily……………..……………………………..2
C. more than once daily…...……………………………...3

Sum to next page:

	Fett	Fiber
Sum from previous page :	☐	☐
	minus	
	☐	
**Total sum point**:	☐	☐

17. I eat fish

A as most once monthly……………………….…0
B a few times monthly…..……………………...2
C once weekly…...……………………………...4
D at least twice daily…………………………...6

18. I eat breakfast

A daily
B a few times weekly
C more seldom or never

19. I eat three (3) main meals per day

A daily
B some times weekly
C once weekly
D a few times monthly
E more seldom or never

What does your usual, daily breakfast consist of:

____

____

____

<u>Fiber point:</u>

17-20 very good
12-16 rather good
6-11 too low

<u>Fat point: Calculated in 2000 kcal.</u>

<40 rather small amounts of fat
40-50 good.
51-71 too much fat
>71 quite too much

Study protocol Diabetes, Metabolomics, and Microbiota

1. Identifying cases with type 2 diabetes from a healthcare center
2. The patients are contacted by a letter, with a question whether they want to participate in the study and information about a phone all within some days.(BO, EL)
3. Patients are called to ask whether they want to be included in the study. When the patients are called, they are screened for inclusion criteria and exclusion criteria

Inclusion criteria:

Age span: 18–70 years
Both parents born in Scandinavia
Type 2 diabetes

Exclusion criteria:

Alcohol and/or drug abuse
Already on ongoing weight-reducing diet
Inability to understand the Swedish language
Major prior gastrointestinal surgery
Pregnancy
Severe food allergy
Severe heart, pulmonary, cardiovascular, malignant or psychiatric diseases
Severe liver disease (spontaneous prothrombin complex (INR) > 1.1)
Severe renal disease (glomerular filtration rate (GFR) < 30 mL/min/1.73 m^2)
Type 1 diabetes

4. If the patient is interested in the study and fulfill the inclusion criteria and no exclusion criteria, a letter is sent home to the patient with further information about the study, a letter to sign for approval to participate in the study, a study questionnaire about lifestyle habits, sociodemographic parameters, and medical history; nutrition questionnaire; SF-36 short-form questionnaire; and visual analog scale for irritable bowel syndrome (VAS-IBS) to be completed before the first visit. Instructions were also included on how to sample feces and tubes for feces samples.
5. Visit 1 at the hospital.
    a. The study questionnaires are delivered from the patients (study questionnaire about basal characteristics, nutrition questionnaire; SF-36; satiety scoring; VAS-IBS).
    b. After this, the patient meet a doctor (Bodil Ohlsson or Ghassan Darwiche) who controls that all questionnaires are completed correctly. Anamnesis is taken and a basal physical status is performed. Physician protocol is completed. Dietary instructions are given in oral and written form. The patients are given all questionnaires to complete at home after the 12-week intervention (study questionnaire; Nutrition questionnaire, VAS-IBS; SF-36).
    c. Blood, feces and urine sampling
6. Visit 2 at the hospital
    a. Fasting plasma glucose, insulin, proinsulin, anthropometric protocol
7. Visit 3 at hospital
    a. Fasting plasma glucose, insulin, proinsulin, anthropometric protocol
8. Visit 4 at hospital

    a. A meeting with the physician. Collection of all questionnaires and check that all are completed appropriately (study questionnaire; Nutrition questionnaire, VAS-IBS; SF-36)
    b. Blood, feces and urine sampling
    c. The dietary intervention is finished.
9. Visit 5 at hospital
    a. Follow-up after further 4 months weeks with the same protocols and sampling as visit 4.

Analyses

Feces: gut microbiota with 16S RNA. Commercially available in Kiel

Blood samples: plasma metabolomics: Metabolomics in Umeå

Hormone and cytokine analyses in Malmö through ELISA and mesoscale. Standard analyses at Department of Clinical Chemistry, Skåne University Hospital, Malmö.

Overall study questions: To evaluate the clinical effects of a modified diet on metabolic risk factors such as diabetes and obesity, and the mechanisms this nutrition composition exerts on gastrointestinal physiology, inflammatory responses, and quality of life, where the controls are their own controls.

Within this study, we wil be able to relate the nutritional composition to metabolic disorders, the gut microbiota, secreted intestinal neuropeptides, and bio- and inflammatory markers in serum, plasma, and urine

Statistical analysis plan
Traditional statistical analyses (parametric or non-parametric tests, Fisher´s exact test) were intended to use to study basal characteristics, change in symptoms, and laboratory analyses. Bioinformatical analyses for microbiota. Biostatistical analysis for metabolomics.
